# Intramedullary spinal cord metastasis of cholangiocarcinoma: a case report

**DOI:** 10.1186/s13104-015-0998-y

**Published:** 2015-02-14

**Authors:** Laurence Faugeras, Gaetan Cantineau, Jean-Francois Daisne, Thierry Gustin, Lionel D’hondt

**Affiliations:** Department of Medical Oncology, Centre Hospitalier Universitaire Dinant-Godinne, 1 Rue Dr G. Therasse, 5530 Yvoir, Belgium; Department of Radiology, Centre Hospitalier Universitaire DinantMont-Godinne, Yvoir, Belgium; Department of Radiotherapy, Clinique et maternité sainte-Elisabeth, Namur, Belgium; Department of Neurosurgery, Centre Hospitalier Universitaire Dinant-Godinne, Yvoir, Belgium

**Keywords:** Cholangiocarcinoma, Intramedullary spinal cord metastasis, Brain metastasis

## Abstract

**Background:**

Cholangiocarcinomas are rare tumors, and metastasis to the intramedullary spinal cord is also rare. To the best of our knowledge, this is the first case of simultaneous cholangiocarcinoma and intramedullary spinal cord metastasis to be described in the medical literature.

**Case presentation:**

A 62-year-old Caucasian male with a cholangiocarcinoma presented pain around his left shoulder without any other symptoms. The results by magnetic resonance imaging and F18 fluorodeoxyglucose positron emission tomography/computer tomography revealed an intramedullary metastasis at the C4 level, with spinal cord compression, and numerous secondary parenchymal brain metastases.

**Conclusion:**

This patient was treated successfully with a combination of radiotherapy, corticosteroids, and chemotherapy. He experienced complete relief of the symptoms and showed improvements upon subsequent radiological evaluations.

## Background

Cholangiocarcinomas are rare tumors that develop in the bile duct. Their incidence worldwide is <1 out of 100,000 persons per year. The prognosis for patients with cholangiocarcinoma is quite poor; the median survival for patients with unresectable cholangiocarcinoma is <1 year. However, in 67% of patients with extrahepatic cholangiocarcinoma, Whipple’s duodenopancreatectomy is an option that increases the 5-year survival rate from 12% (for patients unable to receive the operation) to 28% for those that undergo the surgery [1]. Central nervous system metastases of cholangiocarcinoma are extremely rare; only a few cases have been reported in the literature. Both meningeal carcinomatosis and brain metastases of cholangiocarcinoma have been described [2-4]. To our knowledge, this study provides the first description of a patient with cholangiocarcinoma that presented with brain metastases and with intramedullary spinal cord metastasis (ISCM).

## Case presentation

**Figure 1:**
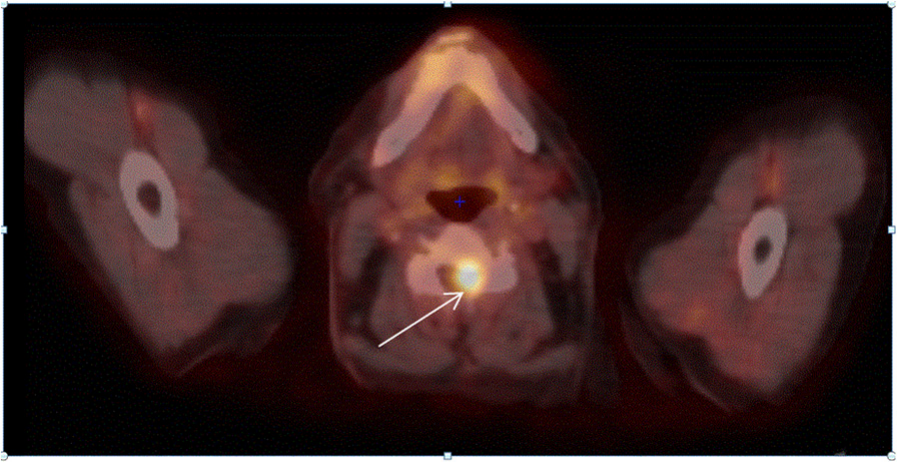
**F18 fluorodeoxyglucose positron emission tomography/computed tomography shows spinal cord intramedullary metastasis of cholangiocarcinoma at the C4 level.** Metabolic activity (standardized uptake values of 6.7 g/ml) was observed in the left paramedian intraductal region (arrow).

**Figure 2:**
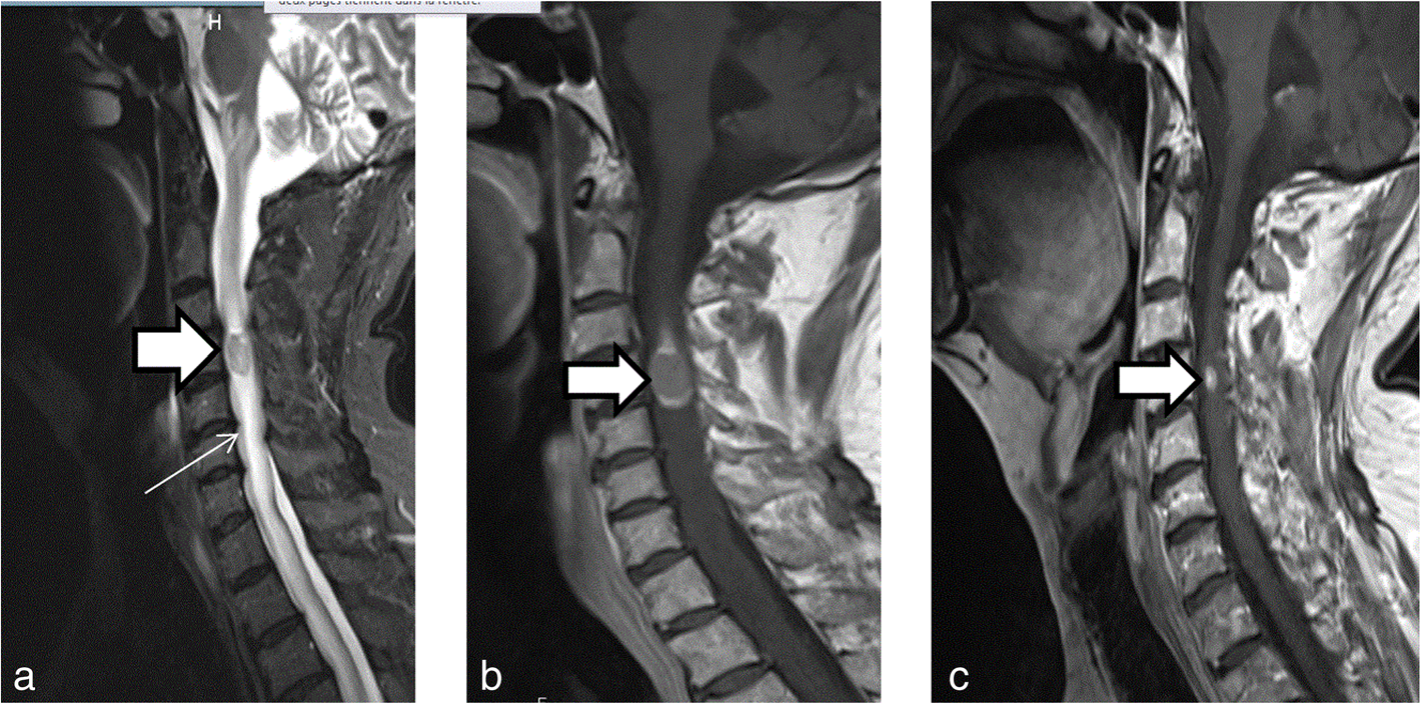
**Magnetic resonance imaging of intramedullary spinal cord metastasis of cholangiocarcinoma. (a)** Magnetic resonance imaging of the cervical spine with T2 short-tau inversion recovery sequences show intramedullary metastases of cholangiocarcinoma at the C4 level. The hyperintensity extended in the spinal cord cranially and caudally relative to the lesion, corresponding to perilesional edema (arrow). **(b)** Magnetic resonance imaging T1 sequences of the cervical spinal cord acquired at the same level as in **(a)**. Images show contrast enhancement around the medullary lesion, which was moderately intense in the central region and at the periphery (arrow). **(c)** Three months after radiotherapy, magnetic resonance imaging T1 sequences show that the intramedullary spinal metastasis had regressed markedly.

**Figure 3:**
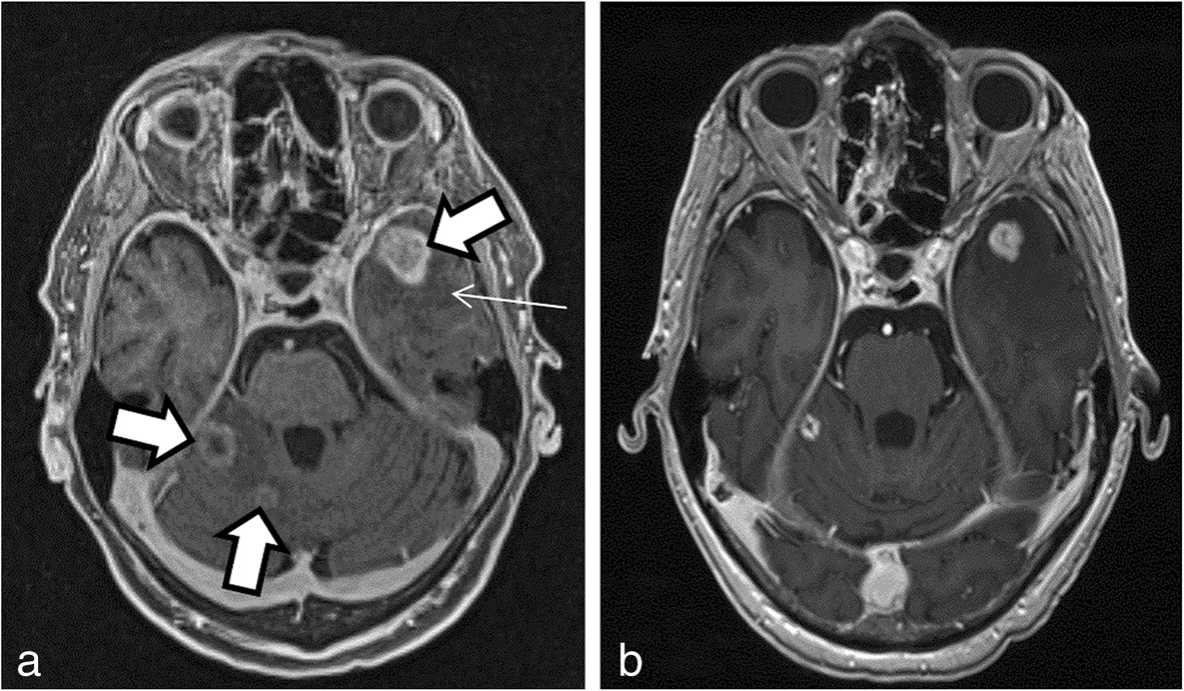
**Magnetic resonance imaging of brain metastases of cholangiocarcinoma. (a)** Multiple metastases of the cholangiocarcinoma were observed before radiotherapy in a T1 multiplanar reconstruction of magnetic resonance imaging sequences after intravenous injection of a gadolinium chelated contrast agent. Rounded lesions are present in the left frontal lobe and right cerebellum. The lesions show peripheral enhancement and are moderately thick and irregular. The lesion center is hypointense or nonenhancing (thick arrows). A perilesional hyposignal corresponding to perilesional edema (thin arrow) is also present. **(b)** Evolution of brain metastases 2 months after the end of radiotherapy, followed by chemotherapy. The lesion volume is reduced, but no changes are apparent in the enhancement features or in the perilesional edema.

Following the appearance of a rapidly evolving jaundice, a 62-year-old Caucasian male was diagnosed with moderately differentiated cholangiocarcinoma localized at the level of the common biliary duct. This tumor was classified as cT3N0M0, or subtype I, according to the Bismuth classification. A surgical resection was performed with Whipple’s duodenocephalic pancreatectomy. Two years later, he presented with postprandial pain, and he had lost approximately 20 kg in 1 year. These symptoms were found to be associated with recurrence in the liver and lung. The patient began chemotherapy with cisplatin and gemcitabine (cisplatin at a dose of 30 mg/m^2^ and gemcitabine at 1,000 mg/m^2^ on days 1 and 8, respectively, every 3 weeks). Given the excellent therapeutic response after nine courses, the patient continued monotherapy with gemcitabine alone. Despite the stability of persistent lesions in the liver and lymph nodes, this medication was discontinued, due to the appearance of a discrete pulmonary infiltrate, which suggested gemcitabine toxicity. At the 5-month follow-up examination, the patient reported pain around his left shoulder without any other symptoms. A computed tomography (CT) scan showed an increase in the size of a lung nodule. F18 fluorodeoxyglucose (FDG) positron emission tomography (PET)/CT confirmed recurrence of the cholangiocarcinoma. Moreover, there were two other suspicious lesions: one was in the anterior portion of the vertebral body of L2 and the other was located intramedullary at the C4 level (Figure 1). When the patient returned to learn the results, he reported that, within the last 36 h, he had developed a motor deficit in his left arm. The pain in the upper part of the left shoulder had also persisted. Therefore, magnetic resonance imaging (MRI) was performed, and the results confirmed an intramedullary metastasis at the C4 level with spinal cord compression (Figure 2). The MRI results also revealed numerous parenchymal brain metastases (Figure 3). The high number of brain metastases disqualified the patient for neurosurgery. In addition, the risk of operating on the spinal lesion was considered too high for either a complete resection of the lesion or a decompressive cranial laminectomy. The next day, radiotherapy of the whole brain and cervical spine to C5 was started, combined with corticosteroid therapy (64 mg methylprednisolone/day). A total dose of 30 Gy was administered in daily fractions of 3 Gy, dispensed with two lateral photon beams shaped with a multi-leaf collimator. After completion of the radiotherapy, systemic treatment with cisplatin and gemcitabine was reinitiated, due to the patient’s previous excellent response to this chemotherapy regimen. The clinical evolution of the symptoms rapidly became favorable in terms of managing pain and neurological deficit. By the middle of the radiotherapy treatment, the patient had completely recovered from the paresis in the left upper extremity. At the time of publication of the present study, he remained on chemotherapy without any signs of neurological or systemic progression.

## Discussion

A careful search of the literature for brain metastasis of cholangiocarcinoma did not reveal any cases similar to the one described herein. Therefore, this case study was the first to describe cholangiocarcinoma with intramedullary metastasis.

The occurrence of ISCM is rare; ISCM represents 4.2–8.5% of tumors in the central nervous system and <5% of spinal cord tumors. ISCM occurs in only 0.1–2.1% of all patients with lung cancer [5,6]. The most common cancers associated with this type of lesion are lung cancer (half of the cases, particularly the small-cell subtype) [5,6], followed by breast cancer [5-7], and infrequently, renal carcinoma, melanoma, lymphoma, and colorectal and ovarian adenocarcinomas. The most common location of ISCM is cervical, in 41% of cases [5,6], followed by lumbar and thoracic, in 32–38% and 26–34% of cases, respectively [5,6]. These lesions are probably most frequently observed in the cervical region because the cervix has large vessels, and there is a local abundance of blood vessels [5].

When ISCMs are diagnosed, brain metastases are found concurrently in 41–89% of cases [5-7]. Thus, it is recommended that, when finalizing the work-up for ISCM, clinicians should carry out a cerebral MRI to search for metastases in the brain.

In most cases of ISCM, patients present with a history of weakness, loss of sensitivity, or sphincter disorder. More rarely, other symptoms, such as back pain, radicular pain, or Brown–Séquard syndrome, are encountered. In rare cases, the finding of ISCM is fortuitous (1–8% of patients) [5,8]. When symptoms suggestive of ISCM occur, the diagnosis relies mainly on MRI, which remains the gold standard; MRI detection is superior to detection with myelography and myeloscanning. A recent study by Flanagan et al. showed that 81% of neoplastic lesions of the spinal cord were hypermetabolic, with an average standardized uptake value (SUV) of up to 3.3 g/ml [9]; thus, FDG PET/CT may be helpful for the detection and diagnosis of ISCM. Some cases of ISCM diagnosed with FDG PET/CT were previously described, particularly for tumors of pulmonary, renal, and breast origins [10-13]. Although FDG PET/CT provides less sensitivity than MRI, FDG PET/CT provides the advantage of allowing an assessment of extramedullary disease status.

ISCM may occur via several modes of dissemination. Three different modes have been proposed in the literature [5,10]. The first possible mode of dissemination is through the vascular system. The proposal of hematogenous dissemination was supported by the coexistence of lung and brain metastases, which could result when metastatic agents are transported through the vascular system [5]. A second mechanism for ISCM is carcinomatous meningitis. Tumor cells present in the cerebrospinal fluid (CSF) can infiltrate the vascular Virchow–Robin spaces, enter the spinal cord, and invade the spinal cord parenchyma [5]. The third possible mode of dissemination is transmission by contiguity. Although the dura mater should be protective, there may be a direct passage through the dura mater, via the extradural space of CSF or nerve roots, into the subarachnoid space [5]. The most likely mode of dissemination in our patient was hematogenous, given the simultaneous occurrence of cholangiocarcinoma and brain metastases, even though the metastatic spread of cholangiocarcinoma typically occurs via the lymphatic system [2].

The classic treatment modalities for ISCM are radiotherapy, surgery, chemotherapy, corticosteroids, or palliative care, when the general status of the patient is highly altered. Several small case series have supported the notion that surgery provided superior treatment, in terms of improving symptoms and overall survival [5,6]. The surgical option is preferentially adopted when a single lesion is observed and the systemic disease is under control, when the estimated survival is sufficiently long, and when the general condition of the patient is good [6]. In contrast to surgery, radiotherapy can be delivered simultaneously to brain and/or spinal lesions. Chemotherapy appears to be a preferred option for highly chemosensitive tumors, such as lymphomas and small-cell lung cancer [6]. Despite the poor radiosensitivity and chemosensitivity of cholangiocarcinoma [14,15], whole brain and cervical radiotherapy followed by chemotherapy with gemcitabine and cisplatin was an effective treatment in our patient. The patient responded quite well, and at the time of publication, he remained asymptomatic on continuing chemotherapy.

Cholangiocarcinomas are considered to be poorly chemosensitive. However, this statement must be put into perspective, because cholangiocarcinoma is a rare disease, and we lack large-scale studies. Most studies included a limited number of patients, were not randomized, and were not controlled. This case report confirmed that some patients with advanced cholangiocarcinomas can achieve prolonged survival with chemotherapy.

However, overall survival is also determined by the subtype of cholangiocarcinoma. Extrahepatic cholangiocarcinomas are subclassified by phenotype as sclerosing, nodular, or papillary. The papillary variant is most commonly observed in the distal bile duct, and its prognosis is more favorable than those of the two other subtypes. Seventy percent of hilar cholangiocarcinomas are the sclerosing variant [16].

The prognosis of ISCM is extremely poor, with a median survival of 4 months after diagnosis. The median survival increases to 6 months for patients treated with surgery and to 5 months for patients treated with radiotherapy. In patients that cannot be treated, the median survival drops to 1 month from diagnosis [17].

The rising occurrence of cerebral and intramedullary spine metastases of cholangiocarcinomas is probably due to improvements in the overall survival of patients, with the advent of multimodal therapies. In metastatic disease, the median survival is approximately 7–12 months. Recent studies have shown prolonged overall survival and progression-free survival with modern management strategies for this kind of tumor, including radiosurgery, radiation alone, and adjuvant or palliative chemotherapy [14]. The combination of cisplatin and gemcitabine remains the first choice for chemotherapy in palliative situations [17].

## Conclusions

Cholangiocarcinomas are rare tumors; their metastases into the brain are even rarer. This study described a patient with cerebral metastasis of a cholangiocarcinoma and ISCM. To our knowledge, this study was the first to describe this type of case. The prognosis for this type of cancer is extremely poor. However, our patient was treated successfully with a combination of radiotherapy, corticosteroid, and chemotherapy. He achieved complete relief of symptoms and showed improvements upon subsequent radiological evaluations.

## Consent

The patient provided written informed consent for publication of this case report and any accompanying images. A copy of the written consent is available for review by the Editor-in-Chief of this journal.

## Abbreviations

ISCM: Intramedullary spinal cord metastasis
CT: Computed tomography
FDG: F18 fluorodeoxyglucose
PET: Positron emission tomography
MRI: Magnetic resonance imaging
SUV: Standardized uptake value
CSF: Cerebrospinal fluid
